# Technological Ecosystems That Support People With Disabilities: Multiple Case Studies

**DOI:** 10.3389/fpsyg.2021.633175

**Published:** 2021-02-25

**Authors:** Maria Soledad Ramirez-Montoya, Paloma Anton-Ares, Javier Monzon-Gonzalez

**Affiliations:** ^1^Department of Education, Tecnologico de Monterrey, Monterrey, Mexico; ^2^Department of Education, Universidad Complutense de Madrid, Madrid, Spain; ^3^Department of Didactics and School Organization, Universidad del Pais Vasco, San Sebastian, Spain

**Keywords:** technological ecosystems, educational innovation, case studies, higher education, research

## Abstract

Advances in technology, research development, and teaching practices have brought improvements in the training, levels of autonomy, and quality of life of people who need support and resources appropriate to their circumstances of disability. This article focuses on empirically analyzing the usefulness of treatments that have been supported by technology to answer the question “How do technological ecosystems being used help people with special educational needs?” The multiple case study methodology was used to address six categories of analysis: project data, objectives, processes, outputs and outcomes, technologies, and impact. The processes, open in communication, were characterized as transversal, ethical, and sustainable. The results yielded various technological ecosystems that support people with disabilities, deliver the help they need to improve their health, and provide enjoyable user experiences. At the same time, they promote the training and improvement of teaching methodologies and involve families in order to improve their knowledge, attitudes, and care of children, young people, and adults with functional diversity.

## Introduction

Knowledge management works best through learning models in an inclusive and participatory society, where technologies offer substantial support to ecosystems that link software, hardware, and people. Technological ecosystems are sets of people and software components relating to each other in a physical environment that supports information flows (García-Holgado and García-Peñalvo, 2018). The opening of access to knowledge is important for ecosystems to have a greater impact on learning, information, and research (García-Holgado and García-Peñalvo, 2019; Ramírez-Montoya, 2020). Technological ecosystems exist in various sectors, including health, society, and education.

Technological ecosystems in the medical field have improved the quality of life of people with disabilities, helping with diagnosis, rehabilitation, and access to medicines. Harlaar and Lankhorst (1998) state that technology can systematize information about disabilities, facilitating the physician’s access to such information for better diagnoses and decision-making. Elsaesser and Bauer (2012) argue that technology enables relevant data to be gathered to improve rehabilitative interventions. For example, the Assistive Technology Service Method, the Assistive Technology Device Classification, and the Person and Technology Matching provide an evidence-based, standardized, and internationally comparable framework for effective interventions that are satisfactory to end-users. In this regard, Wuang et al. (2011) identified that technology positively affects individuals in rehabilitation. In a study of children with Down syndrome, the authors observed that virtual reality in Wii games proved beneficial in improving sensory and motor functions, compared to traditional rehabilitation. In another study, Façanha et al. (2019), seeing that people with vision problems may have difficulty accessing medications, worked on mobile applications that helped them identify medications. Such assistive technology has led to direct or indirect improvements in the medical conditions of disabled people.

In the integration of technologies in environments with people in special education, it is important to consider the characteristics that occur in various contexts, depending on the location. There are differences in the schooling of children and young people with disabilities; sometimes they are located in specific centers, special education centers, or an ordinary center, in which case they are considered as special needs – the needs are contextual and are special to the extent that they are not foreseen by the schooling modality (Warnock, 1978). In Nigeria, Iliya and Ononiwu (2020) identified that contextual factors, such as personal, social, and environmental factors, enable or hinder the empowerment of people with disabilities. In addition to these contextual factors, there are management systems with the use of technologies for the care of people with disabilities. In Tanzania, Lwoga et al. (2020) worked with an electronic hospital management information system to improve care for vulnerable patients, help collect important data on disability and maternal health, and improve overall hospital data management, and the findings located improvements in the quality of care for women and people with disabilities. Contextual factors and the use of technologies are presented as key factors in finding possibilities for care.

At various latitudes, efforts are being made to capitalize on the use of technology in the care of people with disabilities. For example, in Taiwan, a social business model for people with disabilities was introduced based on Eden’s mobile service platform for barrier-free transportation, where information and communication technology (ICT) is integrated with transportation service providers and government resources to meet the transportation needs of people with disabilities (Wu et al., 2020). In Spain, the need for ICT training in these environments was located through a study where professionals perceived a low level of training and knowledge of teachers regarding the use of ICT with people with disabilities, so the need to design and implement didactic training to empower teachers was identified (Fernández-Batanero et al., 2020). Similarly, in Australia, they identified that better integration of assistive technology with ICT will improve the quality of people with communication disabilities, and they also found that improved accessibility with affordable high-speed broadband Internet can provide the services that people with disabilities need (Ali et al., 2020). Government support has also been important in bringing technologies closer together as was the case in the Maldives where its importance was identified in improving speech and language therapy services. The government provided financial assistance to persons with disabilities that could be used to access accessible ICT services and parents as agents of service and support delivery (Zahir et al., 2020). However, Setchell et al. (2020) caution that it is important to recognize that well-intentioned attempts to promote the use of ICTs can be counterproductive if they lead to experiences of marginalization and that, to avoid this, inclusive practices could focus beyond access to and the ability to use devices to include considerations of multiple socio-emotional effects. In the search for possibilities for the care of people with disabilities, it is important to locate contextual and social factors.

Social-assistance technological ecosystems seek to improve the interactive capabilities of individuals with disabilities. Cincotti et al. (2008) state that, depending on the type of disability, assistive technology can improve an individual’s communication and mobility. For example, providing brain–computer interfaces in a technological system composed of various devices helps the person perform simple activities independently. However, Pape et al. (2002) argue that the successful integration of technology depends on the individual’s psychosocial and cultural issues. Therefore, it is necessary to explore the meanings they attach to technological devices, their expectations regarding assistive technology, the expected social costs, and the ways of understanding their disability. Along these lines, Riemer-Reiss and Wacker (2000) identified that disabled individuals tend to accept the use of technological support devices when they find a good cost–benefit ratio and have confidence in their ability to help them. The use of assistive technology must be tailored to the disability, context, and cultural conditions of the individual being helped.

Finally, concerning technological ecosystems in the educational field, their design must generate the necessary conditions to guarantee inclusion. Seale et al. (2015) mention that disabled students feel disadvantaged because they have to work harder than other students, managing both their disability and study. Students with disabilities increasingly find themselves in general education settings, so educational institutions must have instructional technology and universal design to develop educational products and services that are usable by individuals with the widest possible range of functional abilities (Edyburn, 2013). Cook and Polgar (2014) state that these designs that include special-needs-education technology should anticipate the person, task, context, environment, inclusion, technological tools, and desired outcome. Brodin and Lindstrand (2003) also report that solutions for special education needs are primarily based on the way teachers understand disability, how it relates to their work, and their digital competencies. Implementing technology in educational environments diminishes the differences that disabled students perceive in their educational processes *versus* other students, so the institution and the teachers must ensure the necessary conditions that render equality of circumstances.

The technological ecosystems helping people with disabilities provide medical, social, and educational assistance. Moser (2006) states that assessing the impact of technology on people with disabilities involves analyzing various enabling and disabling interactions. In the medical field, Alston et al. (2014) state that assistive technology emerged when health professionals advocated for additional resources to help people with disabilities adapt and reintegrate into society after hospitalization. In social services, according to Phillips and Zhao (1993), assistive technology devices help people with disabilities achieve levels of independence that let them participate in society. Finally, concerning the educational field, Burgstahler (2003) states that educational assistive technology is more about universal learning design through electronic and computer technology that facilitates the development of products that everyone can use, whether or not they have a disability.

Technological ecosystems are developed to help people with disabilities according to the conditions of each context. The overall development of technology in daily life facilitates responding to specific needs using common supports such as tablets or smartphones. Therefore, it is increasingly common to talk about different ways to participate, learn, or perform in various contexts, giving way to the concept of functional diversity.

This article focuses on empirically analyzing the usefulness of treatments that rely on technology by answering the question “How do the technological ecosystems help people with special educational needs?” The article starts with a conceptual basis that describes the technological ecosystems that support training for people with special needs. It presents the case study methodology on which this research is based, discussing three case studies in the European community, and closes with an analysis and discussion of the findings and opportunities for further work in the area.

## Materials and Methods

The case study was the method that guided this study. Stake (2007) and Yin (2006) mention that a case study is an integrated, “bounded” system, an object, rather than a process. It is something specific and complex in operation. A case can describe people, institutions, attributes, and relationships. Specifically, in this study, we present cases emanating from research in the European community. The article describes the attributes given in certain studies and analyzes the actions, interactions, scenarios, technologies, and impact.

**FIGURE 1:**
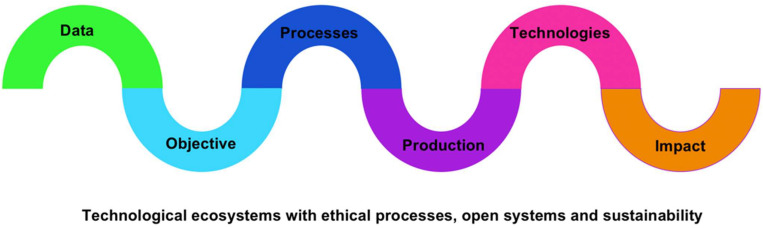
Multiple case study method applied in the study.

The cases can be studied from the particular to the multiple. The description of the cases includes the project data, the background and institutions developing it, the objective, the processes (how it was carried out, its activities, methods, and instruments), production and results (contributions of the project), technologies employed, and impact on specific sectors. Three transversal elements are also described: (a) ethical processes in the research, including ensuring the participants’ privacy, (b) visibility (how the project was communicated), and (c) sustainability (how project continuity is ensured). Figure 1 is the methodology schematic of this research.

The cases presented are Erasmus + KA2, European Union projects in the educational field aimed at teachers’ and students’ training. When the need is detected, it also allocates training and advises other people related to the participants, such as other professionals with various training levels, managers, service personnel, families, and caregivers. It focuses on collaboration and innovation so that knowledge is generated and good practices are exchanged.

These financed projects are designed to have lasting and positive effects in the participating institutions’ communities and are intended to benefit the greatest number of people. The management of most actions is centralized in the Education, Audiovisual, and Culture Executive Agency based in Brussels. Actions are carried out through Alliances for Knowledge, Alliances for Sectorial Competencies, and the strategic associations that are decentralized in the National Agencies. In Spain’s case, these are in the OAPEE, the Autonomous Body for European Educational Programs.

These projects are designed to respond to the need for specialized training, paying attention to functionally diverse students and their specific needs. From a professional point of view, this attention is essential to achieve full inclusion. Organización Naciones Unidas [ONU], 1948; Unesco, 2009; Agencia Europea Para el Desarrollo de la Educación Especial [EADSNE], 2010; Organización Mundial de la Salud [OMS], 2011; Schleicher, 2012; Agencia Europea Para las Necesidades Educativas Especiales y la Inclusión Educativa, 2014; and OECD, 2019; have issued manifests that show the rights of disabled persons and the reality that there is a growing number of these students that must be educated according to their characteristics and needs.

In the projects presented in Table 1, researchers from different fields and universities collaborated and created international, interdisciplinary work teams.

**TABLE 1:** Case studies.

Project title	Financing	Institutions	Duration	Number
DECSA Development of effective coping strategies for vet trainers to provide reliable training to learners affected by psychological disorders	UNIÓN EUROPEA Erasmus Plus Project. 20145 KA2 Strategic Partnerships. Supportive training course and innovation development	4 institutions: Spain, Anadolu – Turkey, Istanbul – Turkey, Bulgaria	Start date: December 27, 2018 Duration: 24 months	2018-1-TR01-KA202-058893
DEMOER The importance of the improved competences of non-formal adult caregivers of elderly people with dementia around Europe and our contribution to their training and support	UNIÓN EUROPEA Erasmus Plus Project. KA2 Strategic Partnerships Erasmus + Programme of the EU Commission Measure: strategic partnership for development of innovation in adult	5 institutions: Spain, Turquia, Bélgica, Chipre, Bulgaria	Start date: November 1, 2019 Duration: 24 months	2019-1-ES01-KA204-063975
INCLUEDUSEX Supportive training course and self-help groups of parents of youth with physical and learning disabilities on sexual education, techniques, and appropriate behavior	UNIÓN EUROPEA Erasmus Plus Project. 20145 KA2 strategic partnerships supportive training course and innovation development	5 institutions: Spain, Bulgaria, Greece, Belgium, Austria	Start date: December 3, 2018 Duration: 24 months	2018-1-ES01-KA204-050062 999874546

## Case Studies


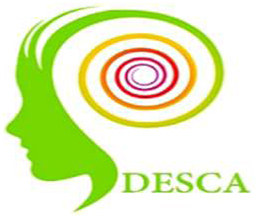


**Case A. DESCA:** Development of Effective Coping Strategies for VET trainers to provide reliable training to learners affected by psychological disorders (DESCA, 2018).

*Participating institutions:* Universidad Complutense de Madrid (UCM), Spain; Kartaltepe Mesleki ve Teknik, Anadolu Lisesi, Turkey, MTAL; Istanbul Milliegitim Mudurlugu, ILMEM; Sdruzenije NA Raboteshtite Uvrezhdaniya, Plovdiv, Bulgaria; NARHU.

*Objective:* To provide training and strategies for teachers who have students with psychological problems.

*Processes:* A preliminary study was done to know the situation in several countries. Observing the lack of training to help students with different problems, the researchers designed the project to detect the current needs. It is a reality that regardless of the severity of their problem, disabled persons are often affected by psychological disorders; they face learning difficulties and require special support. Many trainers do not have sufficient experience and skills to adequately deal with people’s behavioral attitudes and psychological disorders (EUFAMI, 2013). Consequently, they cannot always adopt appropriate responses or use tools and methodologies particular to this population’s needs. This situation causes a feeling of loss of control and great frustration among students and teachers. It undermines the success of the training and the results of the trainers’ work. As part of the preliminary study results, some characteristics of young people with psychological disorders have become known, such as memory and attention span problems. Others include apathy, physical appearance changes, changes in attitude toward teachers, feelings of sadness or depression, confused thinking, reduced ability to concentrate, excessive fears or worries, feelings of guilt, ups and downs, and other mood changes.

*Production and results* of the DESCA project are:

• A manual for vocational trainers who deal with students affected by psychological disorders. It aims to raise awareness about their behavioral characteristics and associated learning difficulties.
• A manual with coping strategies to offer highly reliable training to students with different psychological disorders
• A concept map presenting possible teaching strategies to intervene with people affected by psychological disorders
• Policy recommendation guidelines to improve the professional training of people affected by psychological disorders

The manual is enriched with thematic sections dealing with specific topics related to intervening and treating people affected by psychological disorders in professional training settings. Thematic sheets that focus on specific issues about how to deal with psychological disorders are included. Based on a common general framework in English, the national documents’ content is translated to each partner’s language and adapted with particular references to the partner organization’s context. The final result aims to improve the training of people affected by psychological disorders and facilitate their inclusion both at the professional and social levels. These documents provide knowledge, establish general principles and fundamental legitimate rights, and help overcome the discrimination and stigmatization often suffered by people with disabilities. Miranda de Sousa and Antón (2018) point out that this represents a fundamental paradigm shift to generate new views on integrating this population.

*Technologies:* Technologies were important from the planning through the project’s elaboration phases, starting with the search and then organizing the shared information in Dropbox. Questionnaires were applied and validated, and results were analyzed. Each participating institution created a project webpage with translations to the different languages of the participating countries. Materials, methodologies, videos, and bibliography were presented. Useful contributions for teacher training and socio-educational application are detailed in Gútiez and Antón (2017a, b). Communication was maintained by e-mail, video conferences, team meetings, Gútiez and Antón (2016) and presential international meetings, all according to the established and scheduled plan. The contributions, progress, and analyses presented in these meetings generated comments, debates, and agreements.

*Impact:* Numerous trainers will benefit from the contents to appropriately and sufficiently address the behavioral attitudes of students with psychological disorders. They will have the responsive skills to adapt their methodologies and resources to the needs of this group. They will feel empowered by the acquired knowledge and competencies to intervene in memory and attention problem cases. The trainers will be aware of students’ apathy or reduced ability to concentrate, physical appearance changes, and attitudes toward teachers and classmates. They can observe if the students have feelings of sadness, anxiety, depression, confusion, excessive fears or worries, feelings of guilt, and mood swings. Their awareness and sensitivity will help reduce situations that cause frustration and improve the classroom climate and the results of the teaching–learning process.


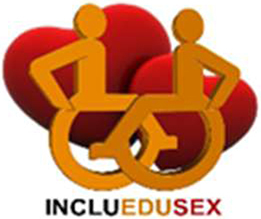


**Case B. INCLUEDUSEX:** Supportive training course on sexual education, techniques, and appropriate behavior, and self-help groups comprised of parents of youths with physical and learning disabilities (INCLUEDUSEX, 2018).

*Participating institutions:* Coordinated by Universidad Complutense de Madrid (UCM), Spain; Austrian Association of Inclusive Society, Vienna Austria; PHOENIXKM BVBA, Kortemark, Belgium; National Association of Professionals Working With People With Disabilities, Plovdiv, Bulgaria; Kotsiras Anastasios-NURBS, Athens, Greece.

*Objectives:* To equip parents with an appropriate sex education program and training materials that enable family members and professionals to contribute to the process of “educating” young people with disabilities to understand and manage their sexuality and express themselves appropriately. To enable parents to guide young people with disabilities to experience full human capabilities. To prevent the potential risks of sexual assault, inappropriate exploitation, sexually transmitted infections/diseases, and unplanned pregnancies at a disproportionately higher rate than the rest of the population.

*Processes:* To bring together families who have physically disabled children to collaborate on improving their quality of life is the project’s objective. After undertaking a needs analysis, we followed the Optional Protocol in the Convention on the Rights of Persons with Disabilities, Organización de Naciones Unidas [ONU] (2006). As primary educators, parents do not provide enough sex education to their children, but parents or guardians should be the first and foremost educators of children’s sexual health. What parents say and do can have a powerful influence on developing healthy sexuality in their children. Most young people with disabilities lack the opportunity to learn about sex and protective behaviors appropriately. It became evident that there is a need to train parents of young people who have some disability to educate them that sexual development is natural, part of becoming an adult, so they gain self-confidence and express their sexuality appropriately.

*Production and results* of the INCLUEDUSEX project were:

• A parental sexual education training course
• A sex education guide to help parents of children with disabilities support professionals in sexual education
• The manual “How to create and manage an autonomous parental group on the subject of inclusive sexual education”
• An inclusive Android-based mobile application offering 24/7 help with solutions to specific queries and problems regarding sex education

*Technologies:* These were a valuable resource before, during, and after the process. Communication was maintained by e-mail, video conferences, team meetings, and presential international meetings according to the established and scheduled plan. The meetings were used to present the contributions, report progress, and generate and propitiate analyses, comments, and debates. The documents and data collected were shared in Dropbox. Questionnaires were applied and validated, and results were analyzed. Each participating institution elaborated a project webpage and translations into the different languages of the participating countries. Due to this project’s characteristics and specificity, 12 short, 2-min videos were created using each participating country’s information and languages: German, Belgian, Bulgarian, Spanish, and Greek. The videos provide information and animated illustrations on different topics (see Table 2).

**TABLE 2:** Videos of the INCLUEDUSEX project.

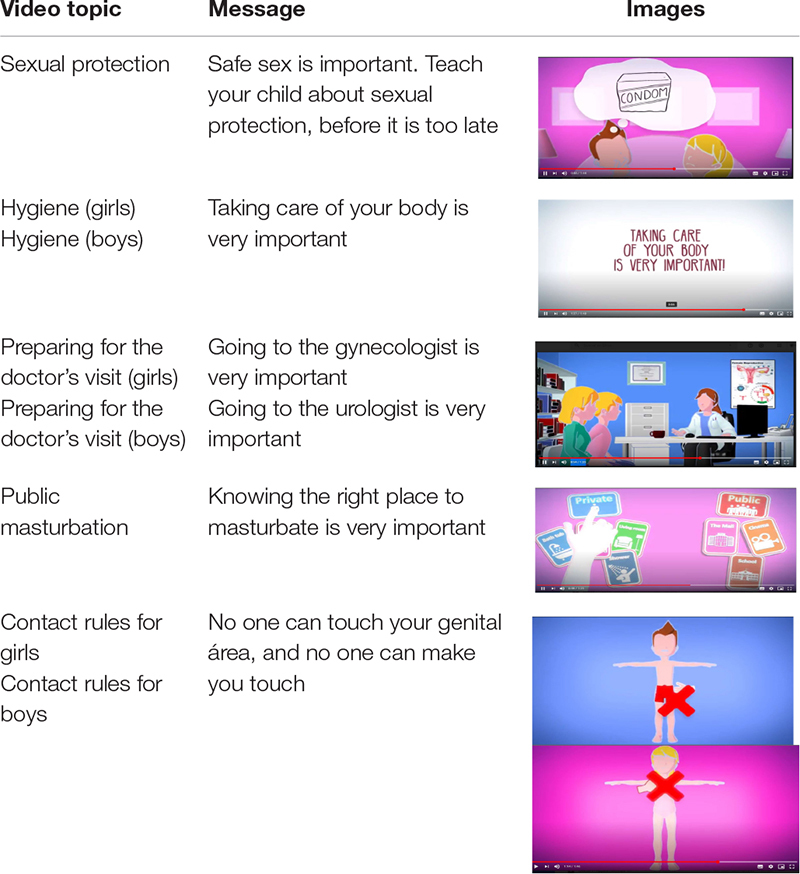

*Impact:* In addition to training young people with disabilities to understand and manage their sexuality and express it appropriately, the parental sexual education training course covered the following types of disabilities:

• Autism spectrum disorders
• Cerebral palsy
• Deafblind
• Intellectual disabilities
• Learning difficulties
• Physical disabilities
• Spina bifida
• Spinal cord injury

The results will provide resources to training professionals, coordinators, developers, parents, and relatives of disabled people, social workers, beneficiaries, children, and youth with disabilities, teachers, NGOs, psychologists, and pedagogues. It is especially relevant to provide sex education to people with disabilities. They are among the most susceptible groups to abuse and need to know how to avoid sexually transmitted diseases and unwanted pregnancies. The project results are useful for professionals who work with disabled people and other relevant-sector professionals. The proposal is innovative and can generate synergies with future studies, projects, and training courses, both for formal and informal education.


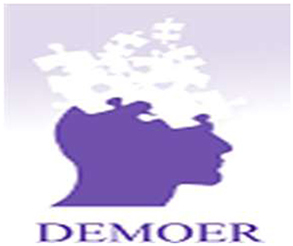


**Case C. DEMOER:** Innovative mobile adult training for family members of people with dementia (DEMOER, 2019).

*Participating institutions:* Universidad Complutense of Madrid. (UCM), Spain; Uluslararasi Sumul Dernegi, Istanbul, Turkey; PhoenixKM BVBA, Kortemark, Belgium; G.M. Eurocy Innovations Ltd., Strovolos Lefkosia, Cyprus; Sdruzenije NA NA Raboteshtite S Hora S Uvrezhdaniya, Plovdiv, Bulgaria.

*Objectives:* To support people in the acquisition and development of basic skills and key competencies; to provide open education and innovative practices in the digital age; to extend and develop the competencies of educators and other staff supporting adult learners.

*Processes:* We started with searches and consultations in publications, international documents, and reference sources. Based on the data obtained and the state-of-the-art analysis, we planned the response to the current needs (OCDE, 2009; OECD, 2019). According to the World Health Organization (Organización Mundial de la Salud [OMS], 2011, 2020), demen- tia is a syndrome that involves the deterioration of memory, intellect, behavior, and ability to perform activities of daily living. Dementia is not an inevitable consequence of aging. The number of people with dementia is increasing rapidly. Alzheimer’s disease, which is the most common form of dementia, accounts for 60 to 70% of cases. Dementia is one of the leading causes of disability and dependence among older people worldwide. Most caregivers of people with dementia are family members. Dementia affects about 50 million people worldwide, about 60% of whom live in low- and middle-income countries. About 10 million new cases are reported each year. It is estimated that between 5 and 8% of the general population aged 60 years or older suffers from dementia at any given time. The total number of people with dementia is expected to reach 82 million in 2,030 and 152 million in 2,050. Much of this increase can be attributed to the fact that low- and middle-income countries are experiencing increasing numbers of dementia cases. Data linked to countries participating in this project indicate that the number of patients with dementia will also increase (estimated at 12% in the next 10 years in Spain and 20% in the next 10 years in Bulgaria and Turkey) as a result of the growing proportion of older people in the population (OECD, 2019). The most common causes of dementia in the European Union are Alzheimer’s disease (about 50–70% of cases) and stroke due to multiple heart attacks or arteriosclerosis (about 30%); other dementia forms include Pick’s disease, Binswanger’s disease, Lewy body dementia, and others.

*Production and results* of the DEMOER project are:

• A guide for adults: “How to support a family member with dementia”
• An open, wizard-style mobile application offering 24/7 help on solutions to specific problems
• An orientation toolkit for adult trainers with methodological support in the delivery of the DEMOER course

**FIGURE 2:**
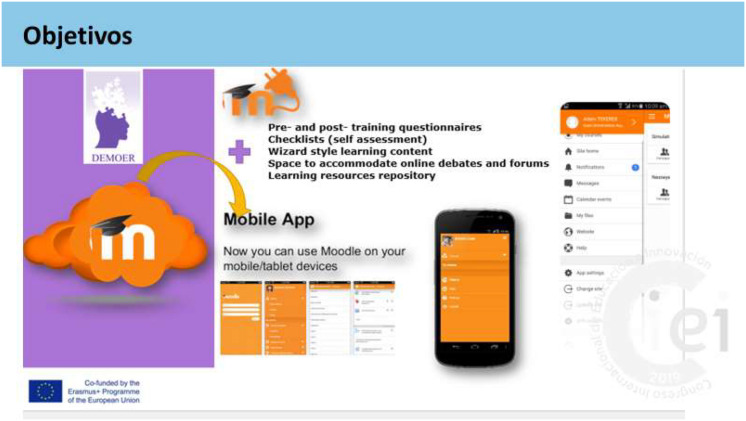
Mobile application for dementia care.

*Technologies:* These were key to developing all the activities involved in the project, from its genesis, progress, and analyses, starting with the search and organizing the shared information in Dropbox. Questionnaires were applied and validated, and results were analyzed. Each participating institution elaborated a project webpage. Work was done in English, and then the information and documentation were translated into the different languages of the participating countries. Communication was maintained by e-mail, video conferences, team meetings, and presential international meetings, all according to the established and scheduled plan. In the different sessions, contributions, progress, comments, and proposals were presented, and a climate of analysis and debate was fostered. In this case, a wizard-style, Android-based mobile application was provided through the learning repository portal. It offers 24/7 help on solutions for each specific problem. The mobile application through the portal serves as a 24/7 resource and training center where adults can study and remember the information contained in the Intellectual Output 1 and 3. The mobile content further integrates the content that the partners already produced in their previous initiatives, namely, the modular content in the P3 Alzheimer’s portal and the P5 DEMOER portal. Based on Moodle, the mobile application also provides the possibility to connect and ask professionals and caregivers questions about the care of a family member with dementia (see Figure 2).

*Participation of adult peers:* The application provides mobile training and allows users to contribute case studies themselves and, after “approval,” share them as useful material; this application is innovative in Europe. The innovation is the adult and peers sharing content, opinions, comments, and materials with other users. All content entered online can be published directly on the mobile application, ensuring easy maintenance and updating. It also allows users to share content with social networks, including Facebook, YouTube, Vimeo, and Twitter.

*Impact:* The greatest impact is the awareness of the importance of providing training and information to family members to understand and provide better support to their family members with dementia. The application includes tips and strategies to use in cases of family member memory loss caused by dementia, which can lead to changed situations and consequences. Cognitive impairment affects memory processes, language, attention, thinking, orientation, calculation, and problem-solving skills. It creates difficulties for the performance of daily life activities like hygiene, clothing, walking, orientation, driving, and eating. It causes behavioral problems like personality changes, bad social behavior, emotional changes, hallucinations and delusions, aggression, depression, and agitation. The manuals support the development of key transversal competencies in the adult population in an informal environment. They provide reliable materials to adult volunteer organizations that support caregivers of people with dementia or those concerned. The manuals are written to be understood easily and facilitate content reflection without any previous medical or psychological background. The availability of online, mobile learning materials with reliable training is a solution to avoid misleading information that often appears on the Internet about the topic. It decreases the emotional and economic burden on patients’ families and helps adult learners (16–29 years old) who take care informally of their family members affected by dementia, trainers and adult educators, and coordinators and directors of volunteer NGOs who provide non-formal care services. The beneficiaries of the improved services are people with dementia, their families, NGO staff, unions of disabled and older adults, and policymakers in the health and social services fields.

### Transversal Processes in the Cases

#### Ethics

The innovative projects are carried out under responsible research principles, preserving people’s privacy and data protection. The values and professional deontology are implicit in all the actions performed, a requirement of great importance: “In this sense, we researchers have to commit ourselves to increase the scientific and professional knowledge of the different aspects that influence training, in the broad sense of the concept. We must assume the responsibility of working with rigor, honesty, integrity, transparency, responsibility, and ethics, favoring studies and results are disseminated and used for professional improvement of people’s lives and, consequently, the betterment of society” (Antón Ares, 2018, p. 40).

#### Dissemination of Results and Open Access

The social benefits derived from research make up a large part of any advanced society’s well-being and improvement. The projects financed by EU Erasmus are available in the Results Platform (European Commission, 2014, 2016, 2020). The database gives open access to descriptions, results, and contact information of projects funded by the Erasmus + program. As indicated on its webpage, inspiration can be found in the set of good practices and success stories, i.e., the projects that distinguished themselves in terms of policy relevance, communication potential, impact, or design. In addition to the Web, other ways that the results and fruits and benefits can be shared include publications, congresses, courses, manuals, videos, and conferences. The channels of open dissemination vary from one project to another. The various participating organizations need to reflect on what types of dissemination are the most appropriate to use and adapt to their level and context to obtain the best use of the activity. The purpose is to enable and expand the benefits of work to improve and modernize education, training, and youth systems.

#### Sustainability

Sustainability means that the financed actions of the developed projects will continue. In Europe, the DEMOER results can be projected to be used during and after project completion to promote lifelong learning in education, training, youth, and sports, supported by European policies. In this sense, dissemination and use are linked, promoting continuity and long-term service and allowing others to benefit from the activities and experiences of the Erasmus + program. The results of each project can serve as an example and inspire other actions sustainable in the future.

## Discussion and Conclusion

This article asked the question “How do the technological ecosystems being used help people with special educational needs?” Three cases were presented of ecosystems that developed and linked software, hardware, and people working collaboratively to provide solutions for people with disabilities. These ecosystems use open-access technologies that have a large-scale impact, with results that support people with disabilities and provide an enjoyable user experience, in addition to helping them improve their health. The results are consistent with authors who advocate for technological ecosystems that integrate software, hardware, open-access technologies, and people (García-Holgado and García-Peñalvo, 2019; Ramírez-Montoya, 2020).

Technologies facilitate the planning, implementation, and dissemination of work designed to improve the training and care of people with disabilities. They enable solutions to be provided for detected needs, adapting to different contexts, and sometimes very diverse environments. They help generate common and specific projects. Moser (2006) invites us to consider the impact of technology on people with disabilities. To know, researchers must analyze the role that technologies play in the various enabling and disabling interactions. In the cases presented, technology helped improve the teaching methodologies, which involved family participation and provided other perspectives, leading to new approaches that contribute to changes in the knowledge, attitude, and attention of children, young people, and adults.

The training and the various content materials allowed the trainers to increase their knowledge, competencies, and sensitivity about the behavior and learning difficulties that disabled students experience, making it possible to implement coping strategies. The acquisition of knowledge helped improve the learning process and avoid school dropout situations due to an incorrect and unadapted implementation of teaching and learning strategies. From this perspective, Cook and Polgar (2014) point out that designs that include special education technology must consider the person, the task, the context, the environment, the technological tools, and the outcome. In the cases presented, the results were developed and disseminated to be adapted to different environments’ needs and transferred to new areas or be useful in influencing future policies and practices. Their results can be maintained after the end of the funding period.

The study identifies some limitations that can be taken into consideration, for example, the differences between the target groups in the different participating countries. There is heterogeneity, all in legislative, institutional, organizational, and cultural aspects, and in other circumstances that affect the fact that the results do not have the same application nor similar effects. In this sense, there are differences in the schooling of children and young people with disabilities; in some cases, they are in specific centers, special education centers, or an ordinary center, in which case they are considered to have special needs; the needs are contextual and are special to the extent that they are not foreseen by the schooling modality (Warnock, 1978). They are seen as a reality which, in turn, through the training and information generated by the intellectual contributions of the research, will be useful to broaden and raise awareness and improve realities and horizons. Another consideration that should be given is the issues related to access to these technologies, considering the social class of children with special needs.

Among the strengths can be placed having clearly and accurately designed the road map, which is of great help. Having established the frequency of meetings and follow-up of the development of the projects also contributes to the sharing of possible problems or complications that may arise and to the search for alternatives and solutions, without losing sight of the objectives. The strength of the results is the variety of recipients and the many people who will benefit as direct recipients as well as their effect. It would be desirable that, on the basis of this work, other projects which complement, extend, and provide new applicable and beneficial knowledge are opened.

The research and training results generated positive changes in the self-esteem, valuation, autonomy, and quality of life of people with some functional disability. They likewise provided resources in different formats, conceived and designed with their needs in mind, processes in which technologies became a valuable resource. They also promoted awareness of people’s needs in vulnerable situations, vulnerable either by one circumstance or another, or their personal characteristics. The case projects considered that their purpose was to improve disabled students’ autonomy and quality of life. Technological advances allow people who have been disenfranchised because of their disability to participate. The general use of these technological resources has also benefited even other users who were not expected to need support. Behind this is the idea of inclusiveness, where considering the needs of a segmented population allows developing more complete proposals that benefit the whole community (Ainscow et al., 2006). There is still a long way to go in terms of technological ecosystems that support people with disabilities. This paper is an invitation to continue collaborating for societal welfare and inclusivity.

## Data Availability Statement

The raw data supporting the conclusions of this article will be made available by the authors, without undue reservation.

## Author Contributions

MR-M contributed to conceptualization, methodology, project administration, writing (final draft), review, and editing. MR-M, PA-A, and JM-G contributed to formal analysis, investigation, and writing (original draft). MR-M and PA-A contributed to funding acquisition. PA-A and JM-G contributed to validation. All authors contributed to the article and approved the submitted version.

## Conflict of Interest

The authors declare that the research was conducted in the absence of any commercial or financial relationships that could be construed as a potential conflict of interest.
